# Pro-apoptotic Bax molecules densely populate the edges of membrane pores

**DOI:** 10.1038/srep27299

**Published:** 2016-06-03

**Authors:** Tomomi Kuwana, Norman H. Olson, William B. Kiosses, Bjoern Peters, Donald D. Newmeyer

**Affiliations:** 1 La Jolla Institute for Allergy and Immunology, 9420 Athena Circle, La Jolla, CA 92037, USA; 2 University of California San Diego Cryo-EM Core Facility, La Jolla, CA 92037, USA

## Abstract

How the pro-apoptotic Bax protein permeabilizes the mitochondrial outer membrane is not fully understood. Previously, using cryo-electron microscopy (cryo-EM), we showed that activated Bax forms large, growing pores. Whether formed in liposomes or in mitochondrial outer membranes, Bax-induced pores exhibit the same morphology, with negative curvature flanking the edges and with no visible protein structure protruding from the membranes. Here we used cryo-EM to show that gold-labeled Bax molecules, after activation by Bid, became localized strictly at pore edges. This argues that Bax acts at short range to deform the membrane. Also, Bax molecules populated the walls of both small and large pores at the same density, implying that Bax is continuously recruited to the pores as they widen. Moreover, because all Bax molecules became oligomerized after membrane insertion, we infer that Bax oligomers are present at pore edges. We suggest that oligomerization may promote pore enlargement.

The Bax and Bak proteins are key effectors of apoptotic cell death. These molecules promote the formation of transmembrane pores in mitochondrial outer membranes (MOMs) and are responsible for MOM permeabilization (MOMP), a critical regulatory step in apoptosis. The resulting breach of membrane integrity leads to the release of pro-apoptotic proteins, such as cytochrome *c* and Smac, into the cytoplasm. These proteins then initiate a caspase proteolytic cascade that promotes rapid cell death^1^,^2^,^3^,^4^. Even if caspases are inactive, MOMP leads to a progressive loss of mitochondrial function that compromises cellular energy production and causes proliferation arrest^5^. Bax and Bak are pro-apoptotic Bcl-2 family proteins^6^,^7^, each consisting of 9 α-helices. Some early structural changes accompanying Bax activation have been identified^8^,^9^. Bax and Bak each contain two α-helices that are amphipathic and become embedded in the membrane after the proteins become activated^10^,^11^,^12^,^13^. However, the detailed molecular structures of fully membrane-integrated forms of Bax and Bak are unknown.

The mechanism of Bax-induced membrane pore formation is also not well understood. A long-standing question concerns the localization of Bax with respect to pore structures. Moreover, it is debated whether Bax/Bak-induced pores are lipidic, i.e. with edges partly formed of lipids, or are circumscribed by a continuous protein lining. Another open question concerns the role of the heterogeneous Bax and Bak oligomers that are invariably formed after full membrane insertion of these proteins^14^,^15^. Recent studies using crosslinking, crystallography and electron spin resonance suggest that these molecules first form dimers^16^,^17^,^18^, which then combine into oligomers. As Bax mutants defective in oligomerization are also generally defective in pore formation^19^, it is commonly assumed that Bax oligomerization is essential for pore-forming activity. However, this has not strictly been proven. Indeed, some recent results have challenged the idea that oligomerization is important for pore formation. For example, a single Bax monomer is sufficient to form a 3.5-nm lipidic pore within an experimental nanodisc structure^20^. Also, we previously showed that the concentration of Bax monomers, rather than oligomers, determines the rate constants for pore formation^14^.

Here, to help resolve such questions, we used cryo-electron microscopy (cryo-EM). Bax-induced pores are not readily observed with traditional EM techniques^21^. However, we showed previously that cryo-EM can capture Bax-induced membrane pore structures, both in liposomes and in isolated mitochondrial outer membrane vesicles (OMVs)^22^,^23^. Here, we imaged Bax localization in liposomes, because OMVs are much less amenable to imaging by cryo-EM. OMVs require thicker ice and cannot be deposited on grids in enough density to be imaged at the high magnifications needed. Nevertheless, imaging of liposomes is informative, firstly because Bax-induced pores in liposomes have the same general morphology as those formed in OMVs^21^,^22^. Secondly, previous studies have shown that Bax-induced liposome permeabilization shares functional features seen in mitochondrial outer membranes, including activation by BH3-only proteins and inhibition by anti-apoptotic Bcl-2 family members^15^,^21^. On the other hand, our previous kinetic studies revealed some features of Bax-induced permeabilization unique to native membranes. These features reflect the presence of ancillary proteins in the MOM, which appear to enhance the intrinsic pore-forming activity of Bax^14^. Nevertheless, activated Bax alone, in the absence of other MOM proteins, can recapitulate the fundamental process of pore formation that occurs during apoptosis.

Our data now show unambiguously that nanogold-labeled Bax became located densely and exclusively at the pore edges, implying that Bax acts at very close range to deform the membrane. Moreover, because we found that essentially all membrane-integrated Bax molecules were recruited into oligomers, we can infer that Bax oligomers are present at pore edges. The data are consistent with the idea that Bax oligomerization could drive the process of pore enlargement and the recruitment of nascently integrated Bax molecules to the pore edges.

## Results

### Nanogold labeling schemes for Bax

**Figure 1:**
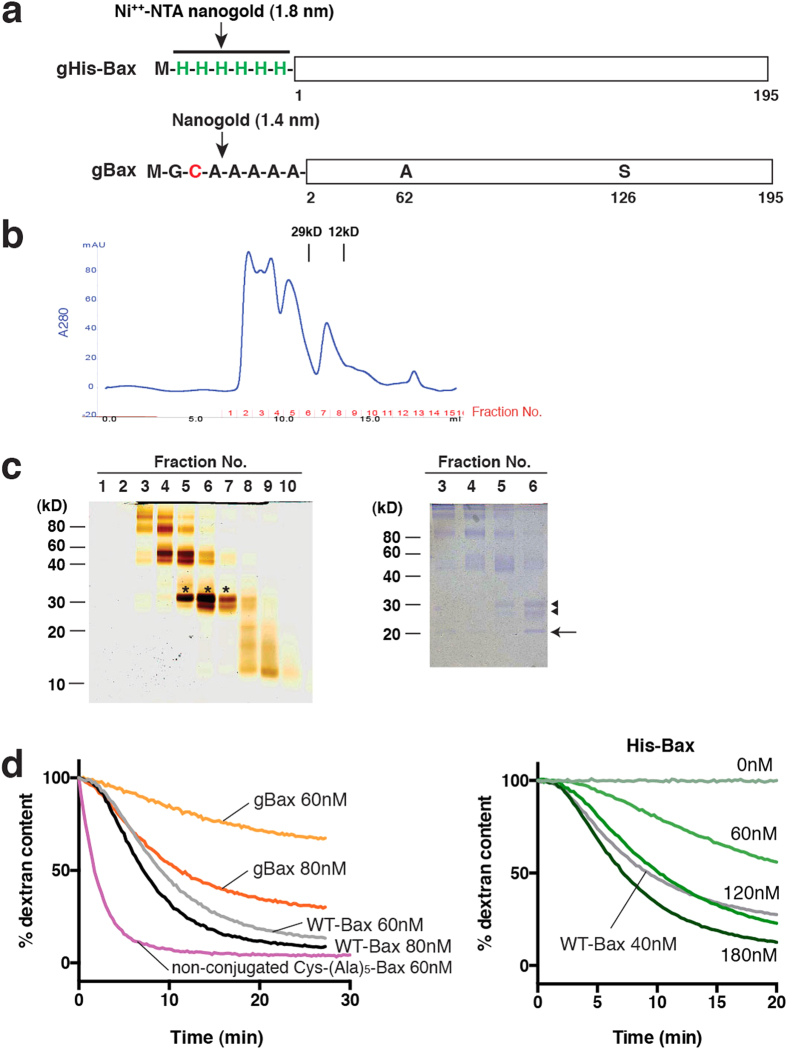
Generation of nanogold-labeled Bax. (**a**) Two Bax constructs used in the study. His-tagged Bax was constructed by inserting 6 histidine residues at the N-terminus of WT-Bax. For direct nanogold labeling, we introduced a single cysteine residue, followed by an alanine linker, near the N-terminus of a Bax construct with mutated internal cysteines (C62A, C126S). (**b**) The absorbance trace at 280 nm from purification of nanogold-labeled Bax on Superdex 75. (Note that the UV trace and the fractions in (**c**) do not match exactly due to the delay volume.) A representative of 4 independent experiments is shown. (**c**) Fraction #6 from size exclusion chromatography shows mostly monomeric and nanogold-labeled Bax. Nanogold in the fractions was stained by silver enhancement on SDS-PAGE (left panel) and proteins were stained with Coomassie Blue (right panel). The samples were loaded without DTT and heat to preserve the nanogold conjugation. Nanogold conjugation shifted the native ~20 kD monomer to an apparent size of 30 kD (*). Coomassie Blue-stained SDS-PAGE of the same samples shows the relative amount of conjugated (arrowhead; 81.4% among the species ranging between 20 and 30 kD by densitometry) and non-conjugated species (arrow). A representative of 4 independent experiments is shown. A lower band was also labeled with nanogold; this species may have been generated by heterogeneity in the nanogold or Bax cleavage during the nanogold conjugation. As the lower band was also nanogold-labeled, the N-terminus of the associated Bax molecule must be intact, suggesting that the C-terminus may be truncated. Such a molecule would be unlikely to participate efficiently in pore formation. (**d**) gBax and His-Bax show reduced activity, measured by the kinetic dextran release assay^14^. Right panel: membrane permeabilizing activity of His-Bax, added with cBid (45 nM). Left panel: membrane permeabilizing activity of gBax or WT-Bax, in the presence of cBid (45 nM). A representative of 3 independent experiments is shown.

To label Bax with gold for cryo-EM localization, we used two approaches, each with different advantages. In the first approach, we added His-tagged Bax (His-Bax) along with cleaved Bid (cBid), to initiate pore formation in cardiolipin-containing liposomes, as described previously for wild-type Bax (WT-Bax)^21^,^23^. Then, we labeled the Bax-containing liposomes with Ni^++^-NTA nanogold (1.8 nm; Fig. 1a). This allowed His-Bax to undergo its activation-induced conformational change and membrane integration without the possibility of hindrance by a bulky nanogold particle. Bax labeled by this scheme is termed “gHis-Bax”. In the other approach, we first conjugated Bax with monomaleimido-nanogold (1.4 nm). We then mixed liposomes with cBid and this nanogold-Bax (gBax), thereby inducing pore formation (Fig. 1a). This direct labeling method avoided a potential complication with His-Bax, in which the 6-Histidine tags on some Bax molecules could become sterically inaccessible to labeling with nanogold particles.

For direct nanogold conjugation to recombinant full-length Bax, we used a cysteine-free Bax mutant (C62A, C126S), which is as active in pore formation as WT-Bax^15^. A single cysteine residue was introduced near the N-terminus, followed by an alanine linker (Fig. 1a). This Cys-(Ala)_5_-Bax construct was labeled by nanogold and purified on a size exclusion column (Fig. 1b). For the labeling reaction, we used a stoichiometric excess of Bax over nanogold, to minimize the presence of free gold particles. This helped ensure that the gold particles seen in cryo-EM images each reflected the presence of a single Bax molecule, although it also meant that some Bax molecules remained unlabeled by nanogold. SDS-PAGE (under the nonreducing conditions needed for detection of gold-labeled species) revealed that nanogold conjugation shifted monomeric Bax from ~20 kD to an apparent size of 30 kD (Fig. 1c left panel, asterisks). Some gBax molecules formed multimers, which were visible in SDS gels (Fig. 1c). To minimize the contribution of these multimers, we chose the fraction with the highest proportion of labeled, monomeric Bax (fraction #6; Fig. 1c). This fraction remained monomeric after concentration and frozen storage (Supplemental Fig. 2). Densitometry analysis of Coomassie Blue-stained gels showed that ~80% of the Bax in this fraction was gold-labeled (Fig. 1c right panel). The Cys-(Ala)_5_-Bax protein (with the two internal cysteine mutations) permeabilized vesicles more rapidly than the wild type protein (Fig. 1d; left lane) and was also stably monomeric.

His-Bax and gBax were both less active than WT-Bax and unlabeled Cys-(Ala)_5_-Bax (Fig. 1d), but they maintained the physiological characteristics of activation by cBid and inhibition by Bcl-xL (Supplemental Fig. 1). This gives confidence that the pores formed by these modified forms of Bax are essentially the same as those formed by WT-Bax. Indeed, we observed pore morphologies that were indistinguishable from those seen in earlier studies with WT-Bax. We added amounts of these Bax proteins that produced sufficient numbers of porated vesicles for cryo-EM imaging. The incubation mixture we used for cryo-EM was substantially different from that used for the dextran release assay, because cryo-EM required a very high concentration of liposomes, which in turn required high concentrations of Bax and cBid for permeabilization. The concentrations of Bax and cBid we used were based on our previous cryo-EM studies of liposome permeabilization^23^, but slightly higher because of the reduced activities of gBax and His-Bax.

### Both gHis-Bax and gBax became distributed around the pore rims

**Figure 2:**
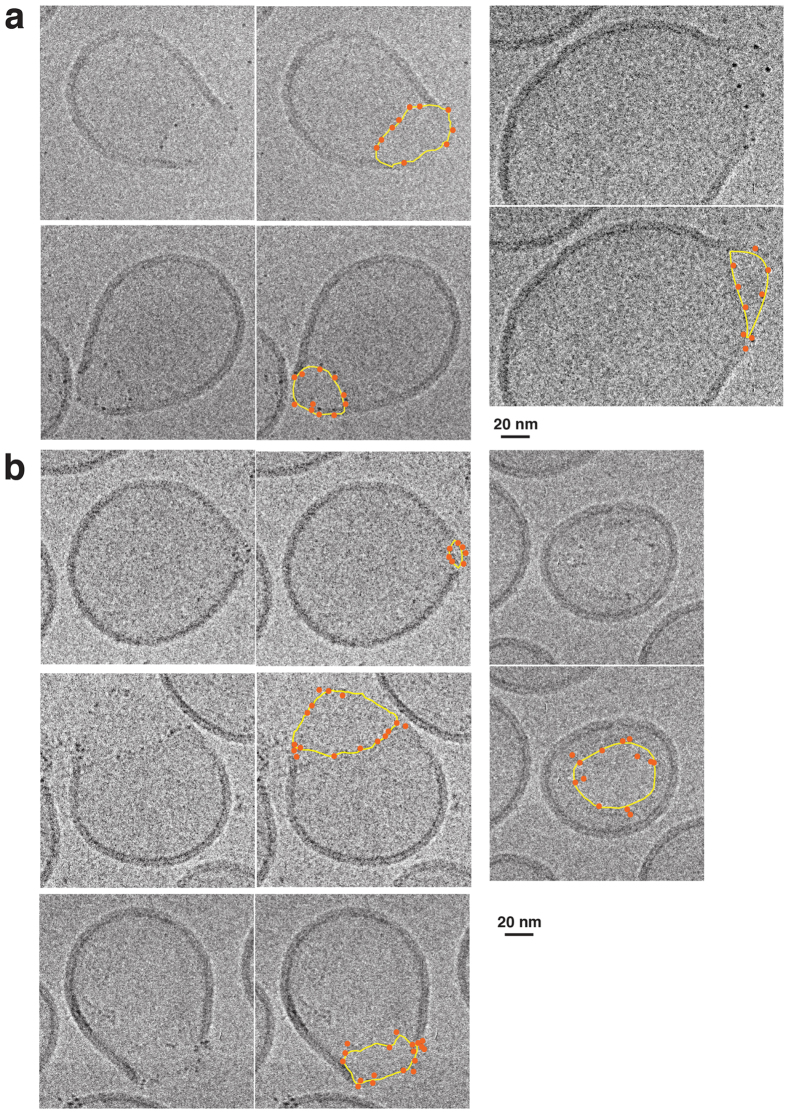
gHis-Bax (a) and gBax (b) were located at the edges of pores. Cryo-EM projection images show that gold-labeled Bax was distributed along the edges of membrane pores. The corresponding images are marked for clarity. Pore edges are outlined in yellow, and gold particles are marked in orange. The markings were drawn manually, for illustration purposes. Three independent experiments were performed. Uncropped images are shown in Supplemental Fig. 5 (gBax) and 6 (gHis-Bax).

With both Bax labeling schemes, cryo-EM showed that gold particles lined the edges of the Bax-induced pores (Fig. 2a,b). With gBax, the small proportion of unlabeled Bax present in the preparations might have played a substantial role in pore formation. However, this complication could not occur with His-Bax, which was the only Bax species present during permeabilization and must therefore have acted in essentially the same way as WT-Bax. As we observed a concordance between the localizations of gBax and gHis-Bax, we conclude that Bax molecules genuinely become located at pore edges. To confirm pore association objectively, we used image analysis. We identified gold particles automatically, by using a size threshold to exclude small debris and by selecting objects within an appropriate pixel density range.

**Figure 3:**
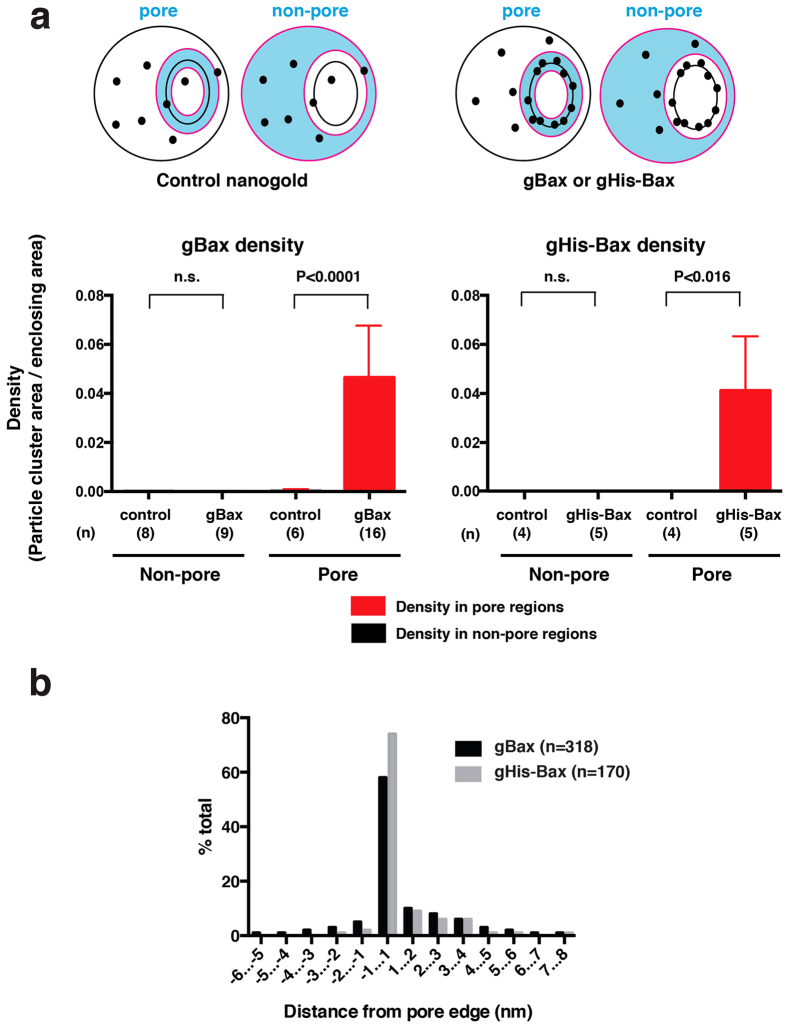
Nanogold-Bax particles were specifically and exclusively associated with pore edges. **(a**) Cartoon showing the pore and non-pore regions of interest (colored in blue) used for image analysis, bounded by the lines in magenta. Image analysis software was used to sum the areas (nm^2^) of nanogold-Bax particles or particle clusters, as well as the total area (nm^2^) of the enclosing regions. Particle density was calculated as the ratio: cluster area/boundary area. For the controls, we permeabilized liposomes with WT-Bax and cBid and added neutralized nanogold (gBax) or Ni^++^-nanogold (gHis-Bax). Data were obtained from a total of n images (n is shown at the bottom of each bar). Statistical significance was determined using the Mann-Whitney test. Note that the density of gBax or gHis-Bax in the non-pore region was not significantly different from that of the control, showing that nanogold-Bax was essentially present only in the pores. (**b**) Distribution of the measured distances between nanogold-Bax and the pore edges. For both gBax (29 pores) and gHis-Bax (15 pores), the gold particles were located predominantly within 1 nm from the pore edges. Because of the foreshortening that would occur in oblique projection images, we chose pores that were oriented approximately en face.

To quantify the localization of gBax and gHis-Bax, we used image analysis to measure the density of gold particles in various regions of interest within liposomes. We manually drew boundary lines enclosing regions associated with pores, or regions outside of pores, then used image analysis software to sum the total area corresponding to gold particles in the outlined regions. As controls for specificity, we permeabilized liposomes with WT-Bax and cBid and then incubated the liposomes with either neutralized nanogold or Ni^++^-nanogold. The results showed that the density of nanogold-labeled Bax associated with non-pore regions was very low and nearly identical to the density produced by the control nanogold particles. In contrast, gBax and gHis-Bax strongly decorated the pore edges. The density of control gold particles was only slightly greater than in the non-pore regions. We conclude that the very low density of Bax-conjugated gold particles located outside pore regions reflected nonspecific association of the gold particles with the membrane (Fig. 3a). In summary, for both gBax and gHis-Bax, association of the gold particles with pores was highly specific, and essentially no particles were found outside the pores.

To quantify the spatial relationships of labeled Bax with pore structures, we analyzed the average distances of gold particles relative to the pore edges. Because the cryo-EM images represented two-dimensional projections of the pores, it was impossible to determine these distances accurately for pores that were oriented obliquely, with respect to the viewing axis. Therefore, we analyzed only the pores that were oriented roughly en face (i.e. that were nearly circular) and which showed visible pore edges. We outlined the pore edges in Adobe Photoshop and then measured the shortest distance between the particle centers to the pore edges, using the ruler tool. The measurements showed that gold-labeled Bax was localized mostly within 1 nm from the pore edges (Fig. 3b). Taken together, the results clearly show that Bax became located almost exclusively and precisely at pore edges.

### The linear density of Bax along the pore rims was independent of pore size

**Figure 4:**
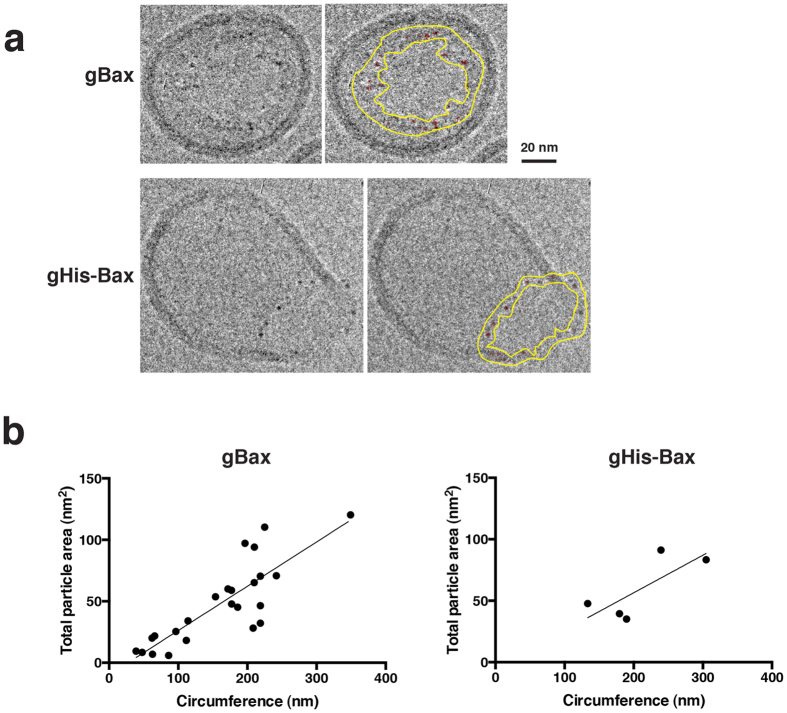
Bax density along the pore edges is the same regardless of the pore size. (**a**) Examples of the pores analyzed. The raw image is shown on left and a corresponding marked image on the right shows the pore boundaries (yellow) and the particles automatically detected therein by image analysis software (red). (**b**) Bax density at the pore edges was independent of pore size, for both gBax and gHis-Bax. The total particle area for each pore was plotted against the pore circumference, which was calculated as the average of the outer and inner boundary line lengths. Because some of the particles overlapped in these projection images, particle area was a more accurate measure of the number of particles than the count of separated particles.

In order to gain mechanistic insight into pore enlargement, we analyzed Bax density along the pore edges. Although these were projection images, the possible error in the measurements due to depth variation was limited, as we observed with side-facing pores that the gold particles were mostly restricted to the plane of the pore. As for our estimates of pore perimeters, any error would also be reasonably low, based on simple geometry. Consider that if a pore has diameter d, its circumference is equal to πd. In the worst case, the measured value of the projected perimeter would occur when the pore is oriented almost edge-on. This would yield a measured perimeter of 2d. Thus, the maximum fractional error is (πd-2d)/πd, or approximately 36%. As the pores we analyzed for Fig. 4b were oriented either en face or at a somewhat oblique angle, as in Fig. 4a, the actual average error in measuring pore circumference would have been much less than 36%.

To measure nanogold-Bax particle density, we drew the outer and inner boundaries flanking the pore rim (Fig. 4a) and used image analysis software to detect electron-dense particles within these boundaries. For both gBax and gHis-Bax, we found that there was a roughly linear relationship between the pore circumference and the total area (in projection) of gold particles associated with the pore rims (Fig. 4b). We conclude that linear Bax density along the pore edges was independent of pore size. This implies that, as pores grow, they must recruit additional Bax molecules. Because the regression lines nearly intersected the origin, the data suggest that the magnitude of Bax recruitment was consistent throughout the entire process of pore enlargement.

To gauge the minimum distances expected between closely spaced Bax molecules, we considered current molecular models, which predict intradimer spacing to be ~2.5 or 5 nm^18^,^24^,^25^. We reasoned that, if Bax densely populated the pore edges, we might observe Bax-conjugated gold particles to be spaced at roughly similar intervals. To test this, we determined the average distance between adjacent particles by calculating the ratio of the pore circumference to the number of particles detected at the pore. On average, we obtained interparticle distances of ~10 nm for gHis-Bax (n = 5) and ~6 nm for gBax (n = 25). These values represent upper bounds for the actual distance between adjacent Bax molecules, because undoubtedly some unlabeled Bax was present in the pores. (For gBax, labeling efficiency was ~80%, but it is unclear whether unlabeled Bax was preferentially incorporated into the pores. For gHis-Bax, the labeling efficiency was unknown, but it must have been reasonably high, as the density of gHis-Bax particles was only slightly lower than that of gBax.) Because these interparticle distances were on the same order of magnitude as those predicted by published dimer models, we conclude that Bax molecules were highly concentrated at the pore edges. However, we note that these cryo-EM images lack the necessary resolution to determine whether Bax molecules form a single or a double ring at the pore periphery. Our data also do not exclude the possibility that portions of the pore wall are free of Bax molecules, as in a proteolipidic (toroidal) pore configuration^26^,^27^. However, considering that not all Bax molecules were labeled with nanogold, the total Bax density must have been greater than that represented by gold particles. It is even possible that Bax molecules were tightly packed at the pore edges.

**Figure 5:**
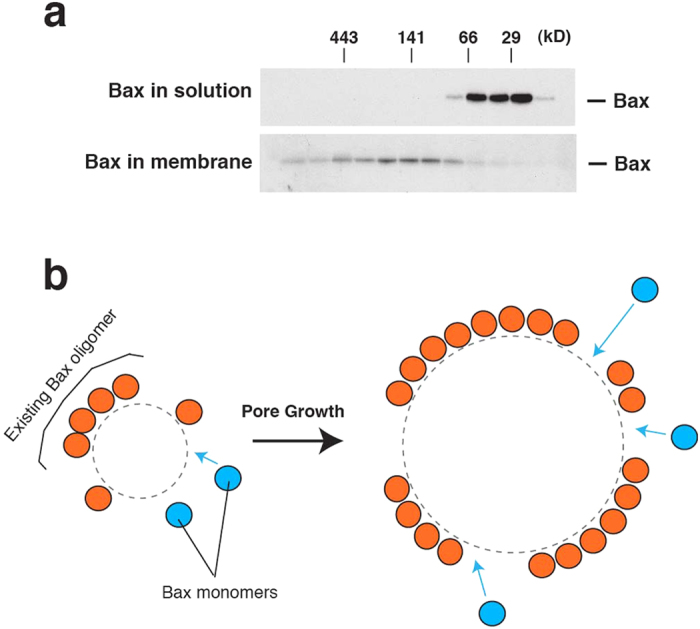
Model of the distribution of Bax oligomers at pores. (**a**) Essentially all Bax molecules became oligomerized upon membrane association. Bax in solution or associated with liposome membranes was analyzed by size-exclusion chromatography. Whereas Bax in solution was predominantly monomeric (upper panel), membrane-associated Bax was almost exclusively oligomeric (lower panel). For membrane-associated Bax, liposomes were incubated with Bax and cBid and collected by float-up centrifugation. The membranes were solubilized in buffer containing CHAPS, and proteins were fractionated on a Superdex 200 size-exclusion column, after which Bax was analyzed by immunoblot. The starting Bax preparation was fractionated in the same way (top). (Bax in solution does not form oligomers in the presence of cBid^21^). (**b**) A simplified hypothetical model of pore growth by Bax oligomerization (viewed from the face of the membrane). New Bax molecules (blue circle) are inserted into the membrane and join an existing Bax oligomer (orange circle) to enlarge the pore (blue arrows). We do not know whether the oligomers extend bidirectionally or whether they form discontinuous clusters along the pore edges. The pore edges are depicted as dashed lines.

Because not all Bax molecules were labeled, it cannot be determined whether a particular Bax-conjugated gold particle corresponds to a Bax monomer, dimer, or oligomer. However, we could analyze the oligomerization status of the bulk population of Bax molecules biochemically, by isolating Bax-permeabilized liposomes and analyzing Bax oligomers in these vesicles by size-exclusion chromatography^21^,^28^. As shown in Fig. 5a, Bax in solution was predominantly monomeric, but membrane-associated Bax was almost exclusively oligomeric. Because our data show that essentially all Bax in liposomes was both associated with pores and also oligomeric, it follows that pore-associated Bax is all oligomerized.

## Discussion

Here we provide the first cryo-EM visualization of Bax molecules in membrane pore structures. Because native MOMs are challenging to image by cryo-EM, especially given the added difficulty of nanogold labeling, in this study we used liposomes. However, we stress that the fundamental mechanism of Bax-induced pore formation is likely to operate in these model membranes, and indeed it has been shown that the regulation of pore formation by Bcl-2 family proteins is well recapitulated in this system^15^,^21^. Moreover, the morphology and enlargement characteristics of Bax-induced pores are the same in liposomes and OMVs^22^. Finally, while this manuscript was being revised, two publications demonstrated by super-resolution fluorescence microscopy in whole cells^29^,^30^ and atomic force microscopy with liposomes^30^ that Bax molecules form large ring-shaped structures. Our results, which use cryo-EM to provide images with higher resolution, are essentially consistent with these reports. We did not observe pores with Bax arranged in arcs, as one of these groups also reported^30^ but rather we found that nanogold-Bax always densely encircled the pores. As the authors of that study noted, their observation of incomplete rings could be the result of limitations of the optical method for imaging pores that are oriented obliquely.

We observed pores with a wide range of diameters and found that Bax was located precisely at the pore rims, at a constant density. We can exclude models of pore enlargement involving a gradient distribution of Bax in the membrane, clusters of Bax molecules at one side of the pore, or multiple concentric rings of Bax molecules. Instead, our results are consistent with the idea that Bax acts precisely at the pore rim to deform the membrane. This proximity of Bax molecules to pore edges is consistent with earlier cryo-EM studies using nanodiscs, which showed that a single integrated Bax molecule could perturb the immediately adjoining membrane, creating a 3.5-nm lipidic pore^20^. Further studies involving multiple Bax molecules in nanodiscs may be able to resolve how additional Bax molecules could assemble to produce larger pores.

### Possible mechanisms of Bax/Bak pore-forming function

Some proteins, such as pneumolysin^31^, are localized at pore edges, but form fixed-size assemblies. In contrast, Bax and Bak belong to a very small set of proteins that can form variable-sized pores. For example, a pro-apoptotic influenza virus mitochondrial protein, sPB1-F2, can form pores of heterogeneous size in planar lipid membranes^32^. Some models for Bax pore formation propose that Bax molecules, by integrating asymmetrically in the bilayer, introduce curvature stress and, at high enough local concentrations, induce membrane perforation^33^,^34^,^35^,^36^. Consistent with this idea, one group showed that intrinsic lipid curvature influences pore formation by Bax-type apoptotic proteins^37^. In this regard, it may be helpful to consider anti-microbial peptides that permeabilize biological membranes, such as melittin (a bee venom), magainin (from Xenopus skin) or mastoparan (a wasp toxin)^38^,^39^,^40^,^41^,^42^,^43^,^44^,^45^,^46^. Significantly, these peptides, 14–26 amino acids in length, form amphipathic α-helices. It is thought that such peptides first bind on the outer leaflet of the membrane bilayer, according to their hydrophobicity sidedness. This induces a mass imbalance between the two leaflets, i.e. a curvature stress, and membrane thinning^39^,^40^,^47^. It is hypothesized that in response to peptide binding, a pore is formed to relieve the stress, as pore formation can expand the interface where the peptides reside^38^,^39^,^40^,^42^,^43^. Helices 5 and 6 in Bax, whose important roles in pore formation are beginning to come to light^12^,^13^,^48^, are similarly amphipathic. Indeed, peptides corresponding to these helices are sufficient to permeabilize membranes^33^. Recent studies have proposed that, in the context of the whole membrane-integrated protein, Bax helices 5 and 6 insert in-plane with the membrane^13^, and in analogy to the pore-forming peptides, they could produce the asymmetry needed for curvature stress.

Despite these features shared between antimicrobial peptides and amphipathic Bax helices, there are important differences in the pores formed by these molecules. For example, early studies highlighted the ability of Bax to cause a large, time-dependent increase in membrane ion conductance^49^,^50^. More recently, we used cryo-EM to provide a physical explanation of Bax-induced membrane permeability, demonstrating that Bax-induced pores grow continuously, with some pores exceeding 100 nm in diameter^22^,^23^. In contrast, the peptide-induced pores typically have a much smaller, fixed size^39^,^42^, e.g. ~8 nm for magainin^51^,^52^. The action of Bax may thus reflect more than the mere similarity of some of its α-helices to antibacterial peptides.

In solution, Bax and some other Bcl-2 family proteins are globular and display a structural fold similar to a class of α-helical proteins, including bacterial colicins and diphtheria toxin, that form protein-conducting pores^53^,^54^,^55^,^56^,^57^. But as colicins form smaller pores, they are not functionally equivalent to Bax and Bak. In summary, the available evidence suggests that the ability of Bax and Bak to form supramolecular pores is not merely a consequence of their sharing the tertiary fold exhibited by colicins and a subset of Bcl-2-family proteins, but must involve other molecular features.

### A potential role for Bax oligomerization in pore enlargement

Taken together with some of our other recent observations, the results we present here allow us to propose a unified view of Bax pore enlargement. We showed earlier that Bax-induced pores in isolated native mitochondrial outer membranes have the same morphology as Bax-induced pores in liposomes^22^. In outer membranes, Bax pores do not have a limiting size, but continue to widen indefinitely. We did not quantify the growth of Bax pores in liposomes over time, but we presume that Bax pores in liposomes also grow indefinitely, as they are permeable to very large macromolecules^21^ and reach similarly large sizes^22^,^23^,^58^. We can reasonably assume that larger pores started out as smaller ones, and thus a snapshot of the pores present at any given time includes pores at different stages of a stochastic growth process.

Once a pore of sufficient size has been opened, Bax molecules could in principle migrate from the outer leaflet of the membrane, across the pore, to the inner leaflet^18^. If so, this would eliminate asymmetry between the membrane leaflets and tend to dissipate membrane curvature stress. We might then predict that the edges of mature pores lack tension. Indeed, we observed that many of the pore edges had a ragged appearance, suggesting a relaxation of line tension. In the absence of tension, how could Bax pores reach diameters exceeding 100 nm, in some cases approaching the entire diameter of the vesicles?^22^ The answer may be suggested by our observation that pores of all sizes contained similar densities of Bax (Fig. 4). We concluded that pore enlargement is accompanied by the continuous recruitment of Bax molecules to the pore edges. This could be the driving force for pore widening.

It has long been assumed, but not proven, that Bax oligomerization is essential for pore-forming activity. Our results now show that Bax oligomers must be present in the pore rims. This follows from two observations: 1) membrane-integrated Bax molecules almost entirely formed oligomers of various lengths (Fig. 5a); and 2) nanogold-labeled Bax molecules were exclusively found at pore edges (Figs 2 and 3). (Although we did not determine whether His-Bax also forms oligomers after it integrates into the membrane, we presume that the small His-tag did not eliminate oligomerization.) Given that Bax oligomers are present in pore edges and that growing pores must recruit new Bax molecules, it is reasonable to imagine that these newly recruited molecules can readily become joined to Bax oligomers already present at the pore edges (Fig. 5b). Hypothetically, oligomerization could assist, or even drive, Bax recruitment to pores. An alternative hypothesis might be that Bax recruitment to pores is merely a consequence of pore enlargement. Such a scenario would require that the existing Bax molecules in a pore at any given time cause a membrane stress sufficient for a degree of pore enlargement. This is perhaps unlikely, especially if the asymmetry between the outer and inner membrane leaflets generated by Bax insertion (this asymmetry is needed to produce curvature stress) becomes dissipated after the initial stage of pore formation.

Paradoxically, our earlier kinetic studies seemed to contradict the importance of Bax oligomers. We found that the rate constants for the permeabilization of MOM vesicles^14^ and liposomes (not shown) are proportional to the concentration of monomeric Bax, i.e. displaying neither cooperativity nor saturation. This implies that Bax monomers, not oligomers, drive the rate of membrane permeabilization. However, it should be noted that the kinetics of biochemical processes are determined primarily by rate-limiting steps, whereas more rapid steps are kinetically “silent”. Bax dimerization and higher order oligomerization could be rapid, non-rate-limiting steps. If so, they would have no effect on MOMP kinetics. Therefore, our earlier kinetic studies do not exclude a role for Bax oligomerization. What are the rate-limiting steps in pore formation? Possibly, these steps could be the membrane integration of Bax monomers and/or the subsequent migration of integrated monomers to the site of a pre-existing pore. If lateral migration were rate-limiting, we might expect to see a substantial amount of Bax located in the membrane, outside the pores. However, we observed very few Bax molecules in non-pore areas (Fig. 3a), and thus, lateral migration of Bax may be relatively rapid. In native MOMs, crowded with proteins, this lateral migration would likely be slower than that in liposomes.

In future studies, it may be informative to analyze Bax mutants that integrate into the membrane but are defective in oligomerization. We did test a Bax double mutant (G108E/S184V) that was reported to possess such characteristics^19^,^59^. Unfortunately, we found that this mutant still displayed substantial membrane-permeabilizing activity (albeit reduced compared to WT) and thus was unsuited for our cryo-EM studies.

### Does Bax oligomerization enhance a feed-forward process in apoptotic MOMP?

What is the purpose of Bax oligomerization? The primary function could be to drive continued pore enlargement, as proposed above. Oligomers might also facilitate the pore-forming process by forming a “sink” for membrane-integrated Bax molecules, thereby lowering the effective concentration of Bax monomers in the membrane and thus biasing the equilibrium towards the integration of Bax monomers and their subsequent recruitment into pores.

Our observation that Bax-induced pores continue to widen indefinitely^22^ suggests that pore growth is a self-sustaining process. Indeed, the Bax BH3 domain, once exposed, can activate other Bax molecules, and the BH3-dependent dimerization subsequently exposes a separate dimerization domain^59^; therefore Bax oligomers could promote their own lengthening. This would make apoptotic MOMP a molecular switch (all-on or all-off) in each mitochondrion. Indeed, MOMP does appear to be an all-or-none event in individual mitochondria. There are situations in which a few, or many, mitochondria within a given cell escape MOMP, depending on their content of anti-apoptotic Bcl-2-family proteins^60^,^61^. The possibility for cells to undergo incomplete mitochondrial permeabilization can enable them, in special circumstances, to escape death after treatment with apoptotic agents. The cells that survive can nevertheless sustain DNA damage, thereby promoting tumorigenesis^61^. These implications for cancer development highlight the need for a deeper understanding of Bax-mediated pore formation.

## Experimental procedures

### Recombinant wild type (WT)-Bax, the mutant Bax for nanogold labeling, His-Bax and cBid

Recombinant proteins were all generated from the vector pTYB1 (New England Biolabs, Ipswich, MA), and the intein tag was removed by DTT treatment according to the manufacturer’s instructions^11^,^21^. As previously described^62^, the Bid construct contained a thrombin cleavage site near the caspase cleavage site, which allowed us to produce a nearly-natural form of cleaved Bid (cBid), in which N- and C-terminal fragments remained associated, similar to caspase-cleaved Bid. Note that the cBid used in this study does not contain the His-tag, unlike the one originally reported^62^. The internal cysteines (C62 and C126) in Bax were mutated to alanine and serine, respectively, by QuickChange site-directed mutagenesis (Agilent, Santa Clara, CA), and an N-terminal extension (MGCAAAAA) was added by PCR. For His-Bax, PCR was used to add six histidines to the N-terminus of wild-type Bax in pTYB1. All the constructs were verified by sequencing.

### Nanogold labeling of Bax

Bax, mutated as described above (280 μg), was pre-treated with mercaptoethylamine hydrochloride (MEA), then 6 nmol of monomaleimido-nanogold (Nanoprobes, Yaphank, NY; #2020A) was added prior to overnight incubation at 4 °C. The mixture was concentrated and loaded onto Superdex 75 equilibrated with 10 mM potassium phosphate, 50 mM KCl and 1 mM EDTA, pH7.4 (KKE buffer). The fractions were run on SDS-PAGE without heat or DTT and stained with Li Silver Enhancement kit (Nanoprobes; #2013) to visualize nanogold, or with Coomassie Blue for protein staining. Nanogold conjugation added ~10 kD to the apparent size of the molecule. As a control, we inactivated the maleimido residue in nanogold with excess ethanolamine in 0.1 M carbonate buffer. The treated nanogold was dialyzed in KKE buffer to remove amine and added to the incubation mixture of liposomes with WT-Bax and cBid. The concentration of nanogold was assessed by absorbance at 420 nm, using a Nanodrop instrument (ND-1000; Nanodrop Technologies, Wilmington, DE). The absorbance value of the control sample was 64.4% of that of gBax, which was used to normalize for the analysis of background (nonspecific) density. Ni^++^-NTA nanogold (1.8 nm) was obtained from Nanoprobes (#2080). Pore-forming activity was measured using a fluorescent dextran release assay^14^. Briefly, we incubated the same liposomes used in the cryo-EM experiments containing 70kD fluorescein-dextran (FD70S; Sigma-Aldrich, St Louis, MO) with various Bax proteins (40–120 nM) and cBid (45 nM) at 37 °C for ~30 min. Full-length Bcl-xL (1.68 μM) was added in some samples. The presence of external anti-FITC antibodies in the assay quenched the released dextrans immediately to monitor the release as decrease in fluorescence signal^14^.

### Liposome permeabilization for cryo-EM

Liposomes containing 7% cardiolipin were generated by a detergent-mediated method^23^. Typically, 2 mg of total lipids (Avanti Polar Lipids, Birmingham, AL) (phosphatidylcholine: phosphatidylethanolamine: phosphatidylinositol: cardiolipin = 55:29:9:7 mol%) were used to prepare ~20 μl of suspension in KKE. 2 μl of liposome suspension were incubated with gBax (4.6 μM; fraction #6) and cBid (3.2 μM) at 37 °C for 2 h. With His-Bax, the liposomes were incubated with His-Bax (3.2 μM) and cBid (3.2 μM). 1 μl of Ni^++^-NTA nanogold was added for the final 30 min of the 2-h incubation. We compensated for the reduced activity of gBax and His-Bax by raising the temperature, protein concentration, and incubation time, to achieve a degree of vesicle permeabilization amenable to inspection at high magnification.

### Cryo-EM

The samples were deposited onto Quantifoil holey grids and plunge-frozen into liquid ethane after being blotted with filter paper^63^. The grids were inspected with a Tecnai G2 electron microscope (FEI, Hillsboro, OR) at 200 kV with ~1.6–3.6 um defocus at nominal magnifications of 50,000x or 29,000x. The images were taken with a CCD camera (Gatan, Inc., Pleasanton, CA).

### Image analysis

The images were imported as calibrated high resolution TIFFs and processed in Image-Pro Premier (Media Cybernetics, Inc. Rockville, MD). We empirically determined that an image intensity threshold range of 30–50, out of 256, appropriately identified gold particles; also a threshold area of 0.32 nm^2^ allowed us to exclude most of the small debris. This threshold was well below the area calculated from the manufacturer’s stated nanogold diameter, 1.3 +/− 0.14 nm (Nanoprobes Inc., personal communication). The particle diameters were stated to fall into a normal distribution, and therefore our threshold (below 4 standard deviations from the mean) would include more than 99% of the particles. To determine whether gold particles were specifically located at pores, we drew regions of interest (ROI) flanking the pore openings and between their inner and outer circumferences (as in Fig. 4a; yellow lines in right panels). Within the boundary of each of the ROIs, the software automatically outlined gold particles (or clusters of them) and calculated the total particle area (a measure of the number of gold particles). We also drew the region within the vesicle boundary, but outside the pore, and applied the same particle detection thresholds. The space outside vesicles was omitted from the analysis, because the debris and irregularities there interfered with particle detection. The density of gold particles (defined as the ratio of particle area to non-particle area) was calculated in the pore and non-pore regions. Twenty-five pored vesicles in 15 images taken from 2 independent experiments were analyzed for the pore region of gBax density and for the non-pore region, 9 images from 2 experiments were used. For control neutralized nanogold, 6 images were analyzed for the pore region and 8 for the non-pore region, from 2 independent experiments. For gHis-Bax, both pore and non-pore regions were analyzed in the same 5 images from 2 independent experiments. For the corresponding control (non His-tagged Bax), the same 4 images from 1 experiment were analyzed for the pore and non-pore regions. Statistical significance was calculated using the Mann-Whitney test.

To determine the linear Bax density at the pore circumference, we chose vesicles (5 from gHis-Bax and 25 from gBax) showing clear pore edges. The particle areas within the outer and inner boundaries of the pores (manually drawn) were detected as described above. To estimate the perimeter of the pore edges, we used the average of the outer and inner pore perimeters.

To measure how closely the gold particles were associated with the pore edges, we selected vesicles oriented apparently en face and showing clear pore edges. Using the ruler tool in Adobe Photoshop, we measured the shortest distance between the center of the gold particles and the edges. It is reasonable to assume that the pore edges are not affected by the presence of the gold particles, as the particles were conjugated beyond the extended linker at the N-terminus in gBax as well as gHis-Bax (Fig. 1a). This segment would be unfolded away from the membrane when it is activated^64^. For gBax, 29 pores were analyzed and for gHis-Bax, 15 pores.

### Detection of Bax oligomers in the liposome

Liposomes were generated by the extrusion method as described^21^ and incubated with Bax (1.8 μM) and cBid (1.26 μM) for 2 h at 37 °C to be permeabilized. After the incubation, the liposomes were floated-up by high-speed centrifugation and collected with microfilters with 0.1 μm pore size. The retained membrane fraction was dissolved in 20 mM HEPES, pH 7.4, 150 mM NaCl containing 1.2% CHAPS and fractionated in Superdex 200 (GE) in the same buffer. Bax alone in buffer was also fractionated. Bax was detected by immunoblotting with anti-Bax antibody (Santa Cruz; N20).

## Additional Information

**How to cite this article**: Kuwana, T. *et al.* Pro-apoptotic Bax molecules densely populate the edges of membrane pores. *Sci. Rep.* **6**, 27299; doi: 10.1038/srep27299 (2016).

## Supplementary Material

Supplementary Information
