# Supplementary material for: Pro-apoptotic Bax molecules densely populate the edges of membrane pores
Source: Sci Rep. 2016 Jun 3;6:27299. doi: 10.1038/srep27299 (PMC4891688; doi:10.1038/srep27299)
Supplement: Supplementary Information [file srep27299-s1.pdf]

Supplemental Material

Pro-apoptotic Bax molecules densely populate the edges of membrane pores

Tomomi Kuwana*, Norman H Olson[1], William B Kiosses, Bjoern Peters, and Donald D Newmeyer

Figures and figure legends

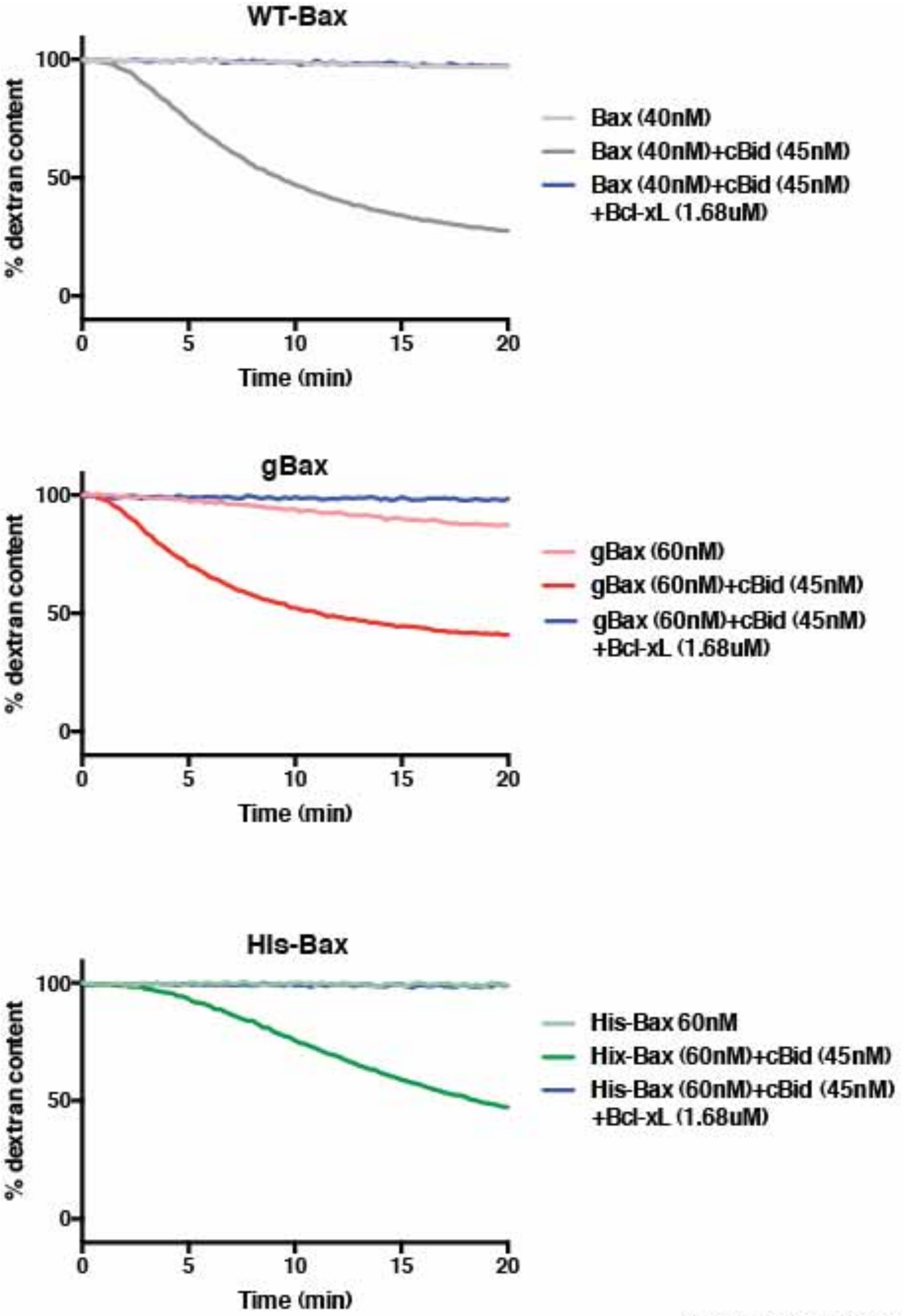

Supplemental Figure 1

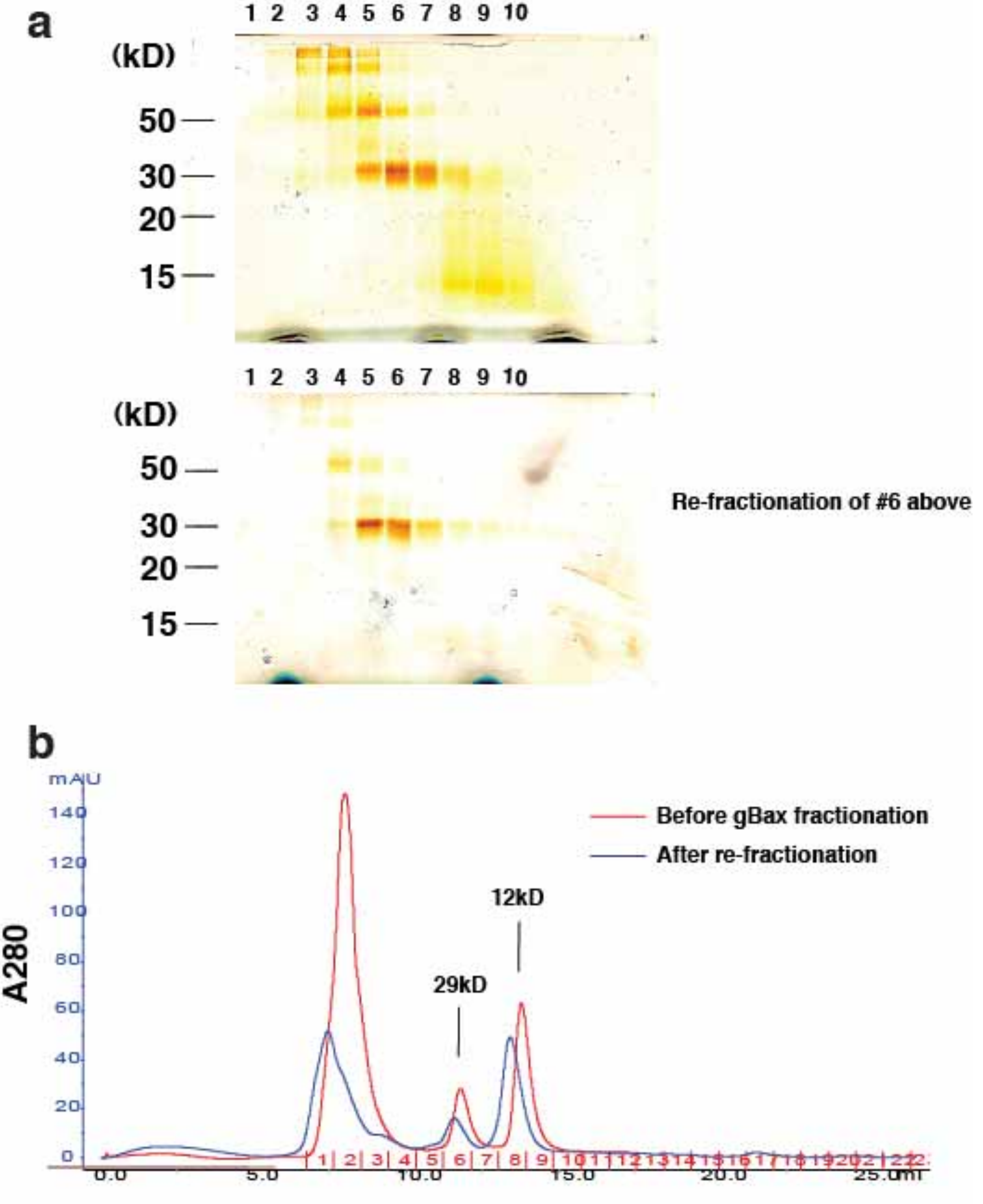

Supplemental Figure 2

a

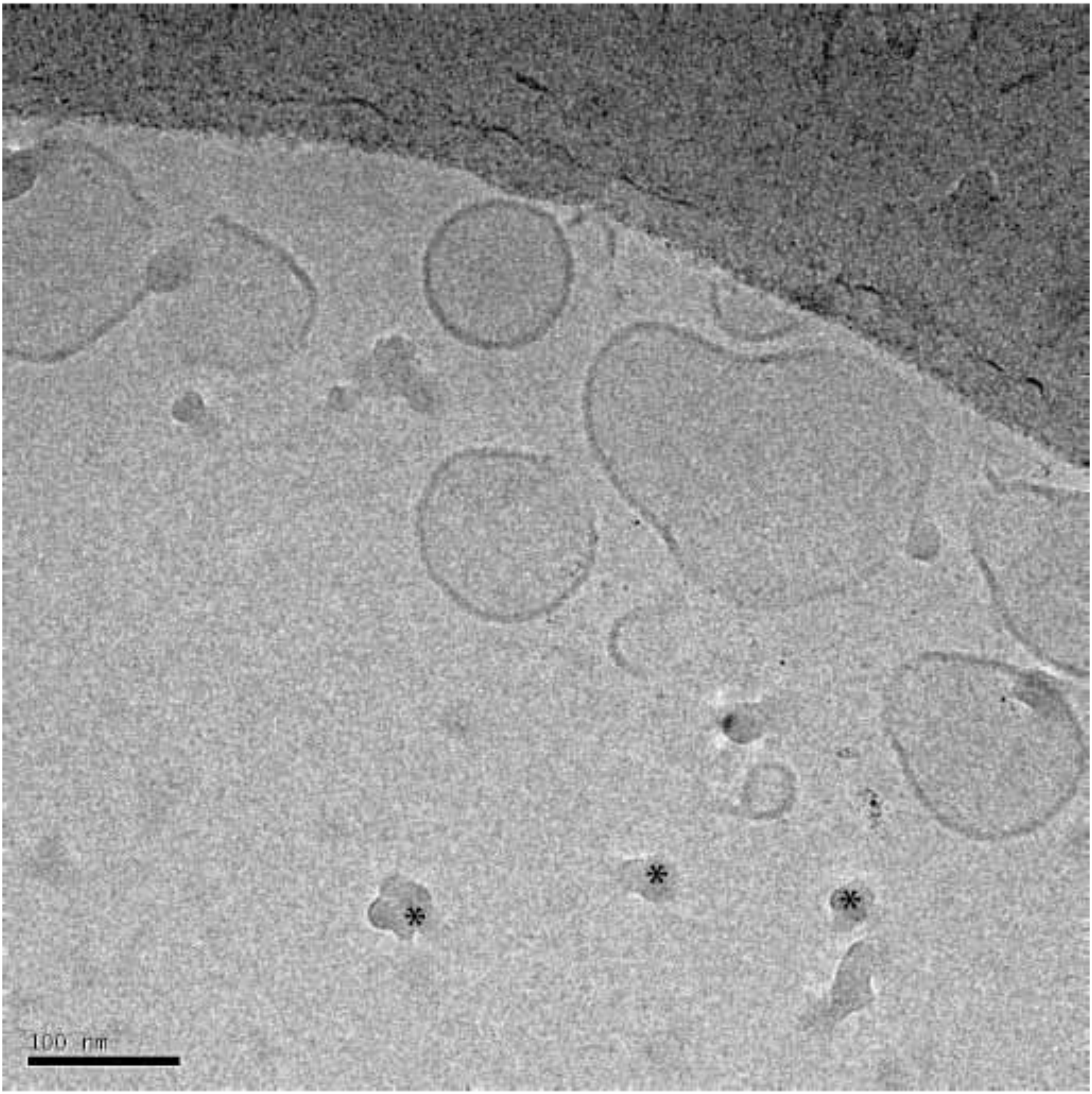

Supplemental Figure 3

b

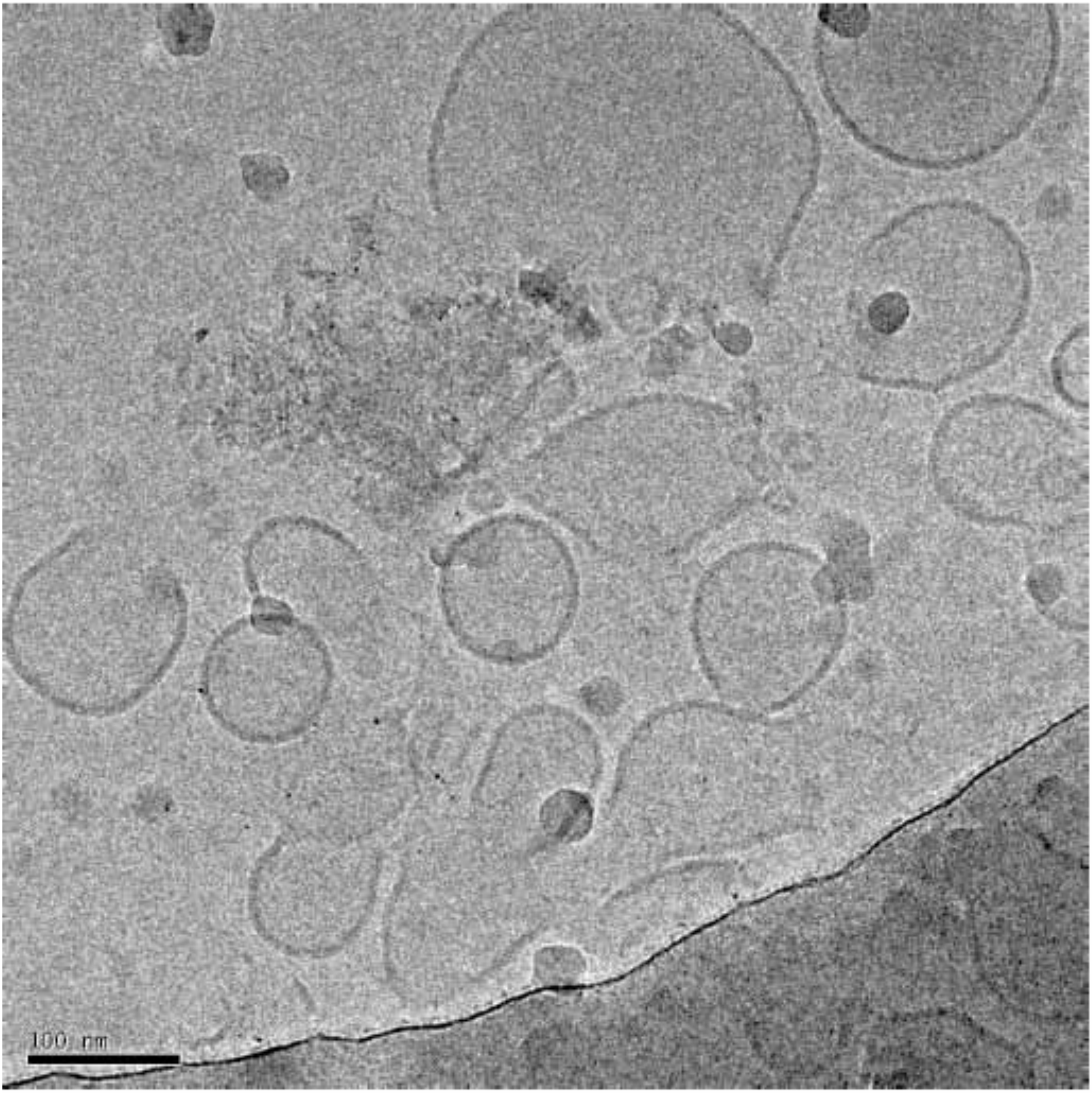

Supplemental Figure 3

c

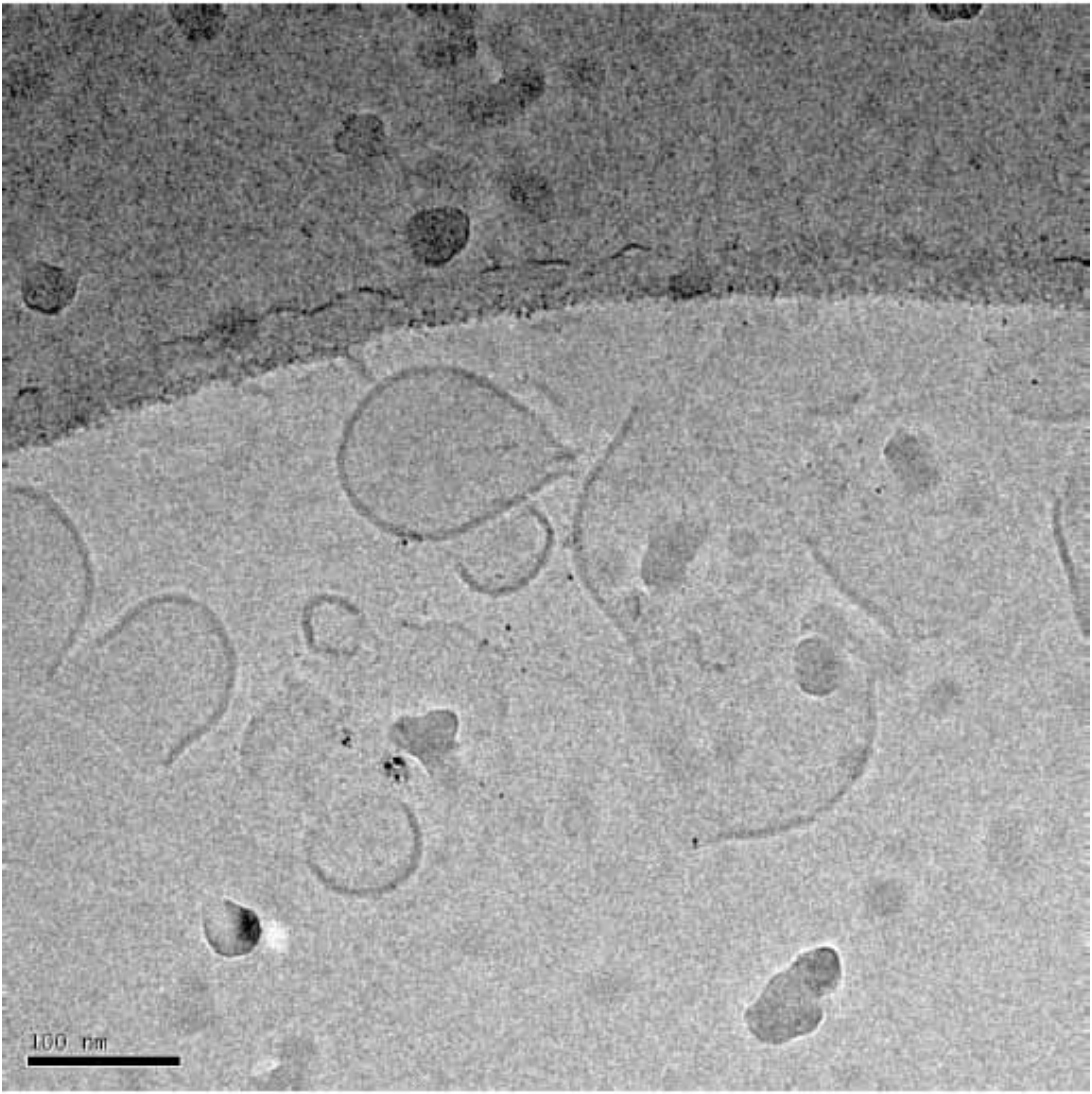

Supplemental Figure 3

a

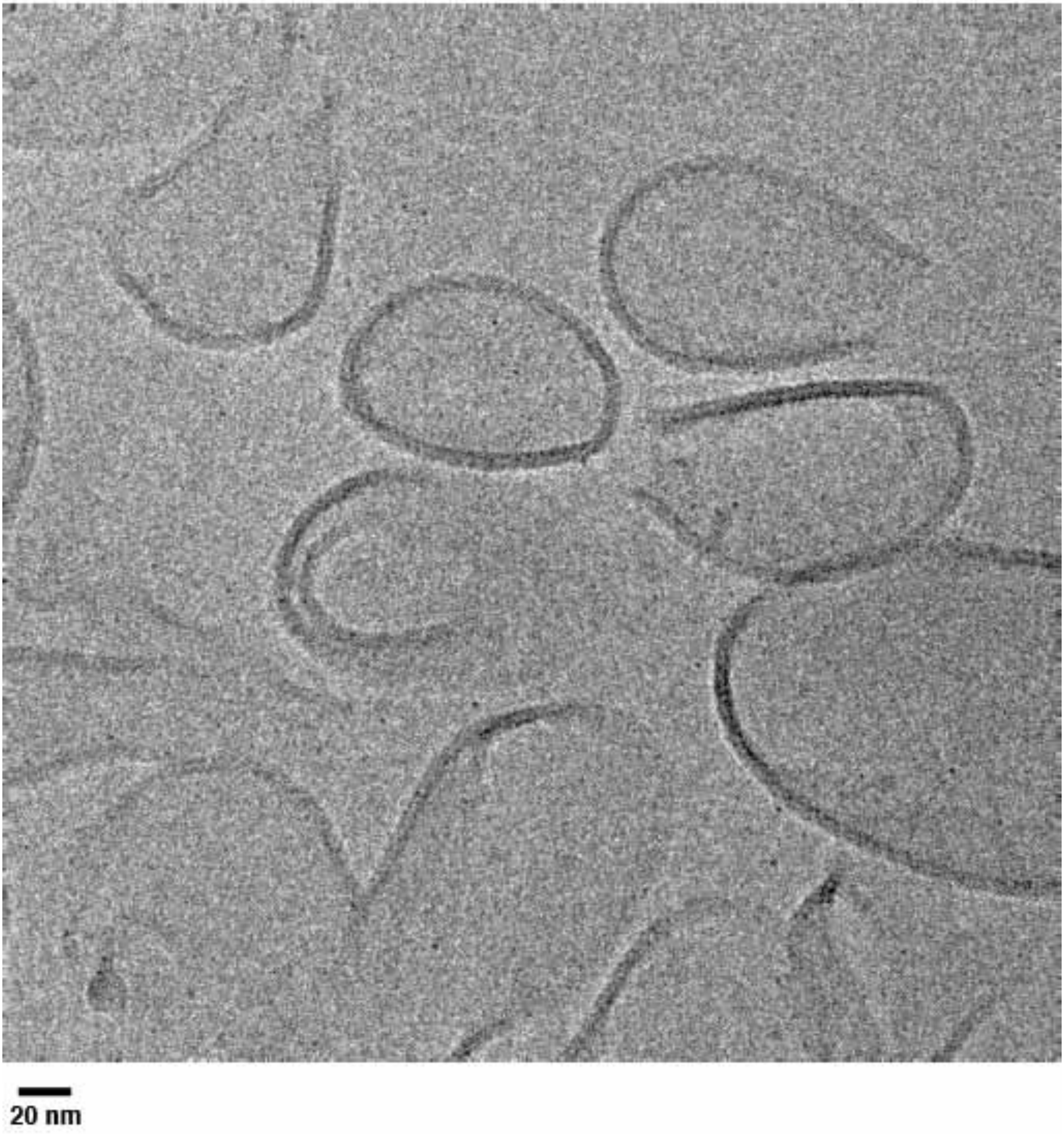

Supplemental Figure 4

b

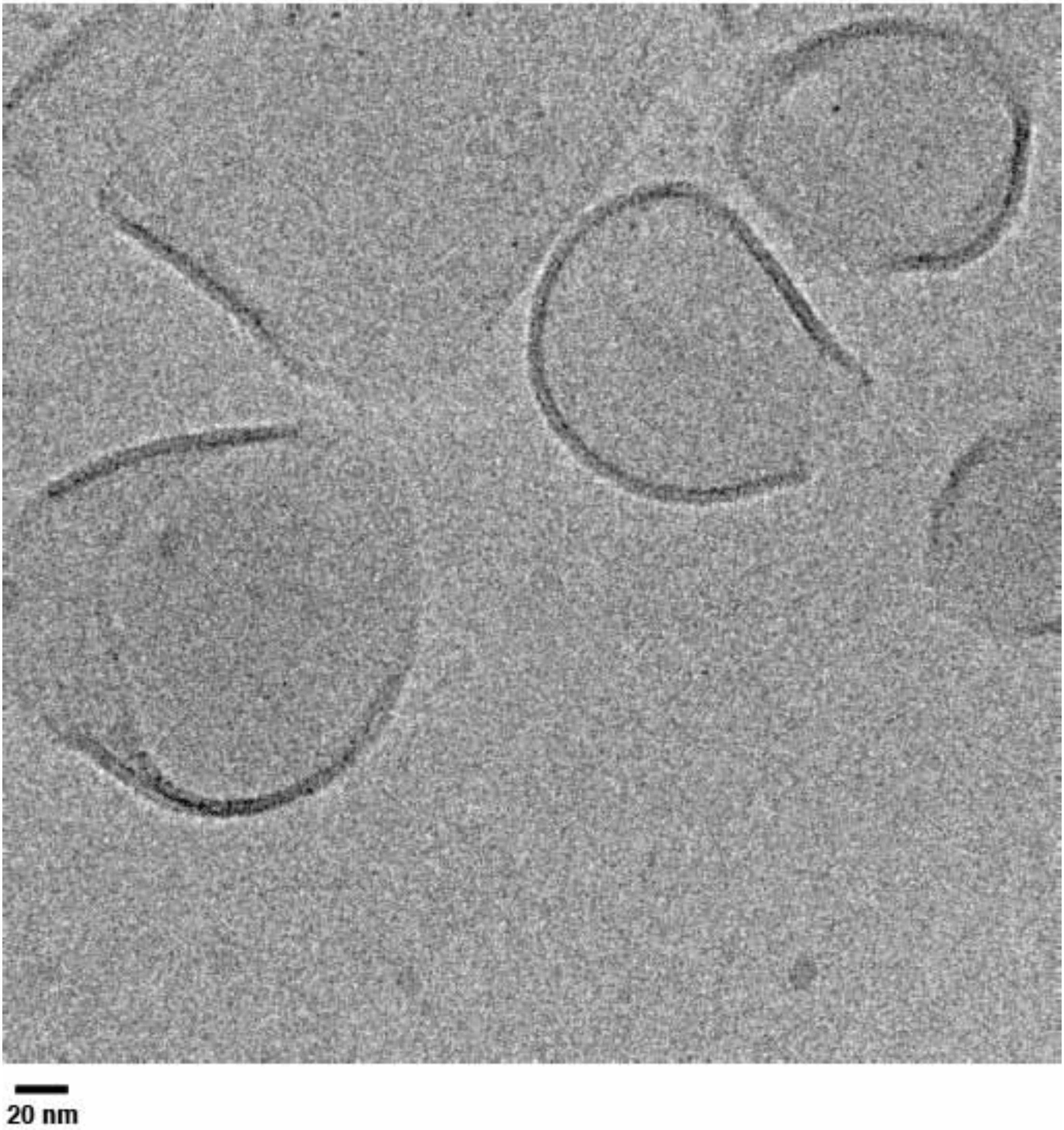

Supplemental Figure 4

c

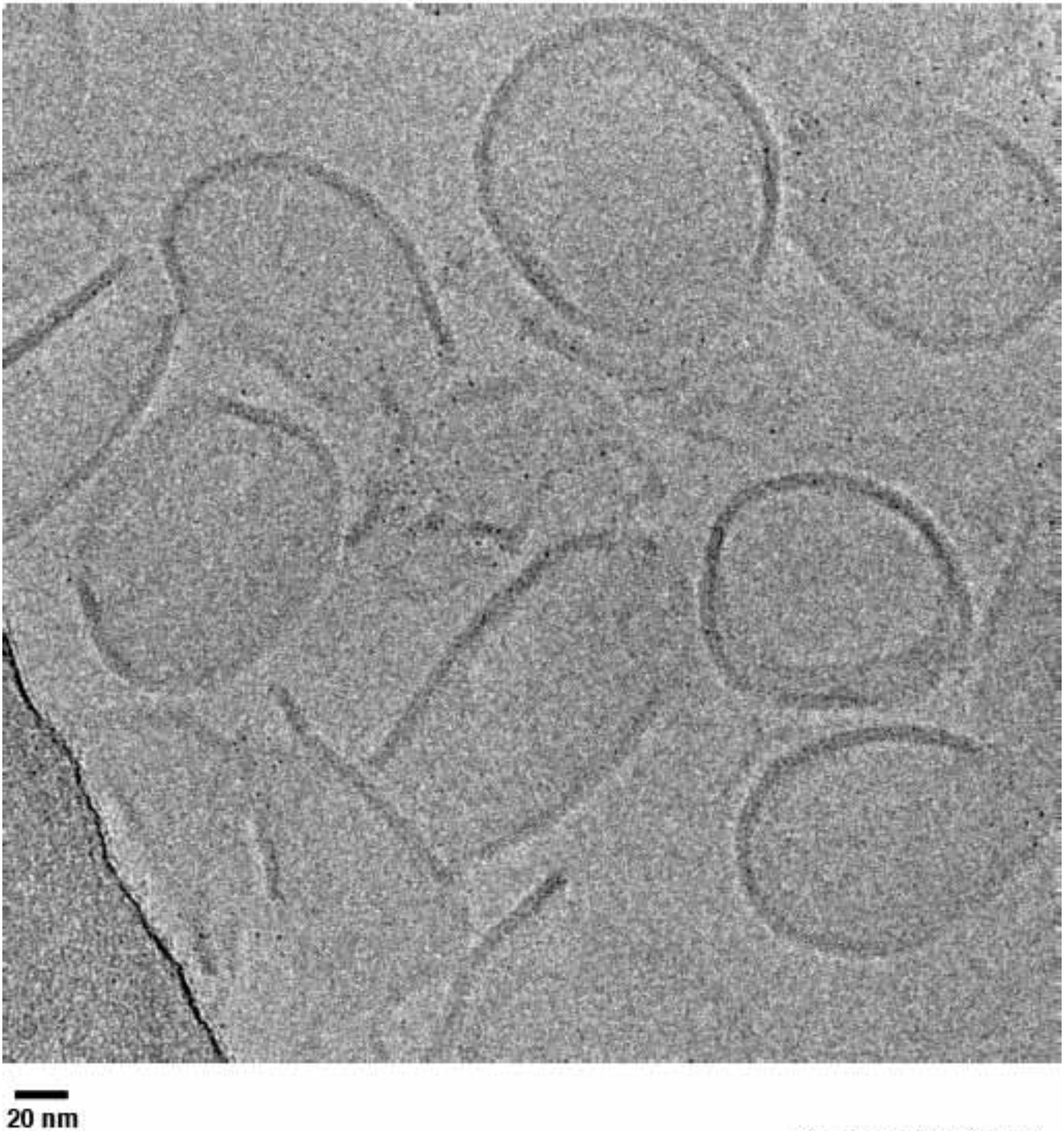

Supplemental Figure 4

a

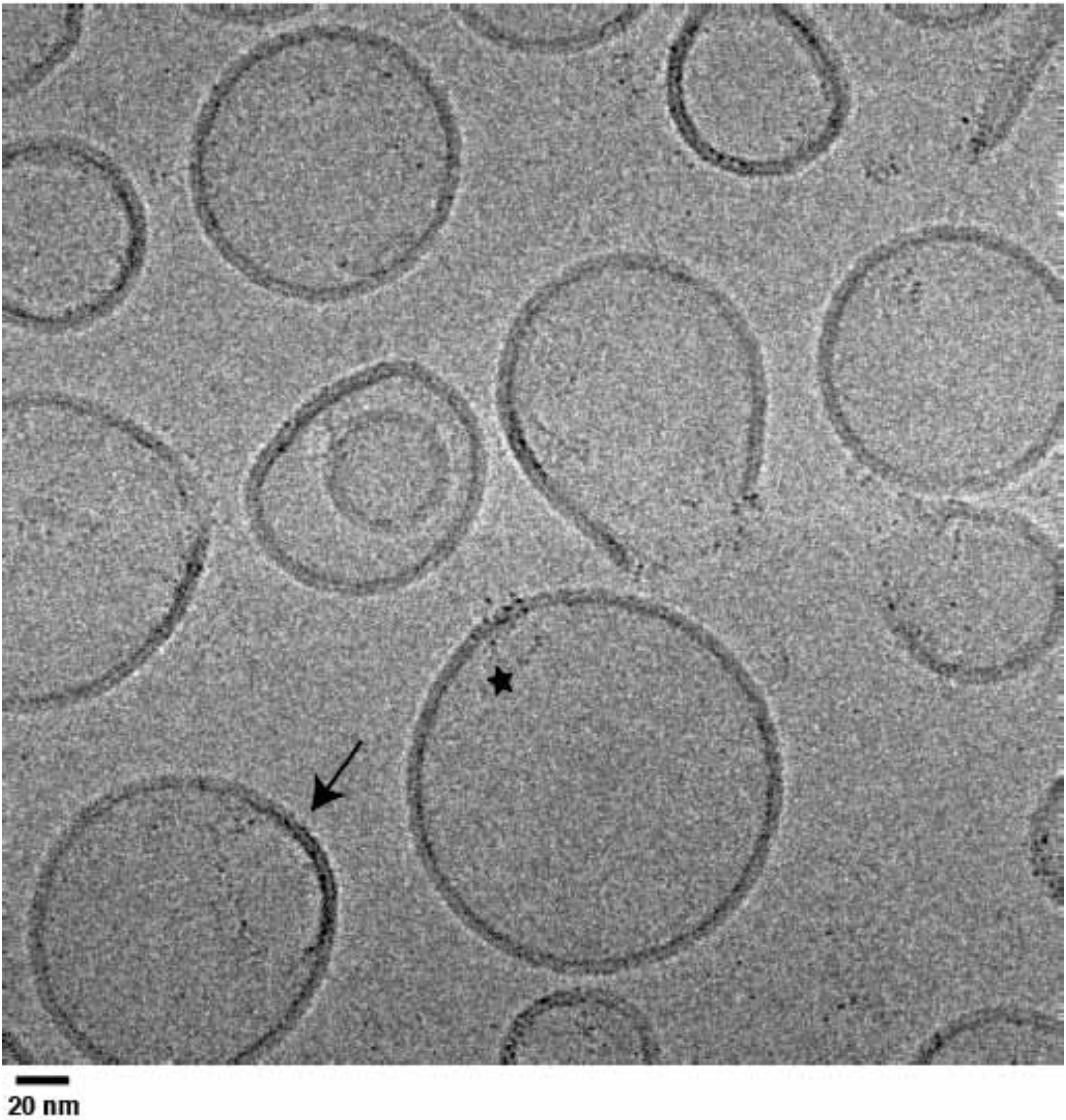

Supplemental Figure 5

b

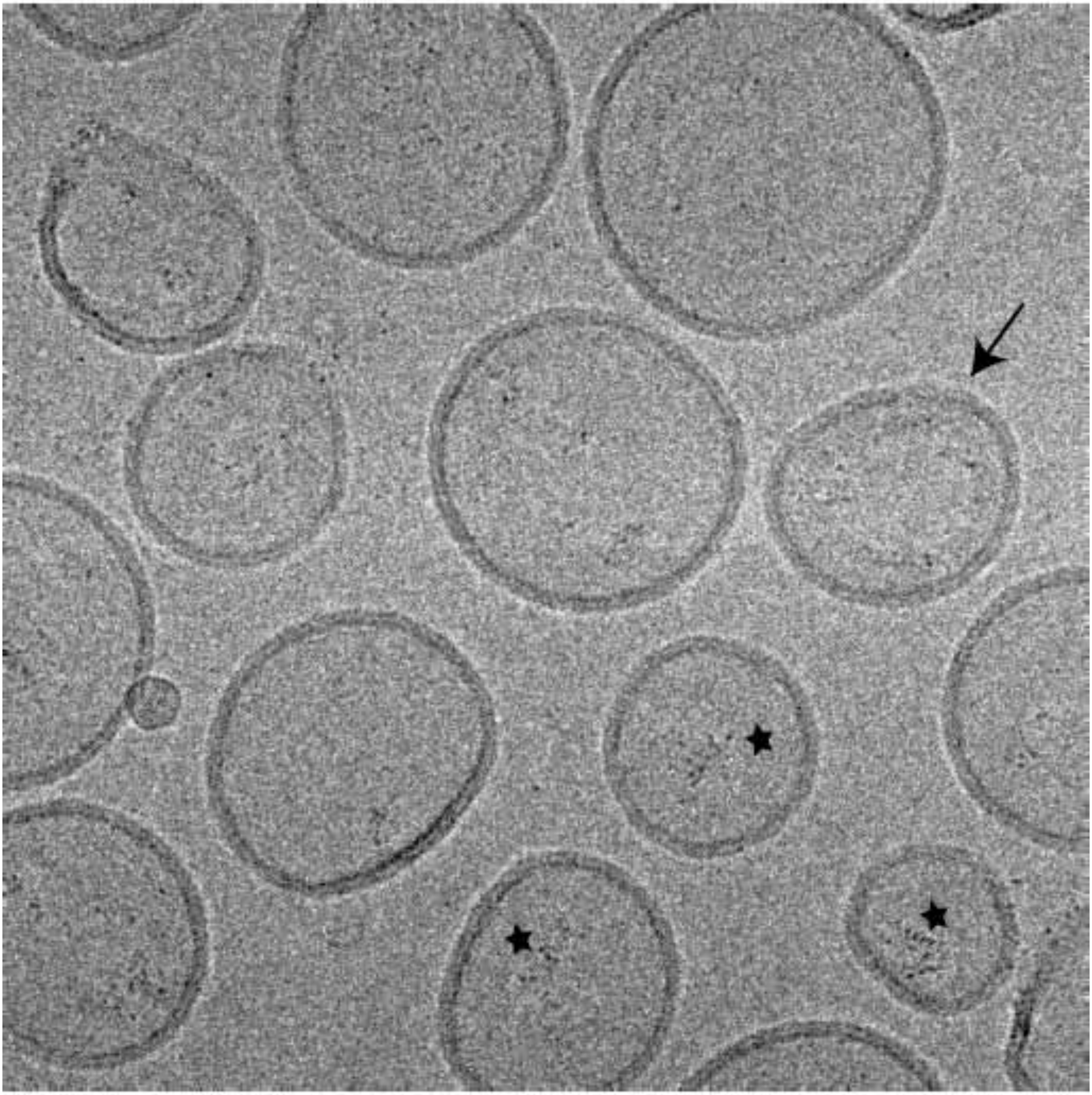

20 nm

Supplemental Figure 5

c

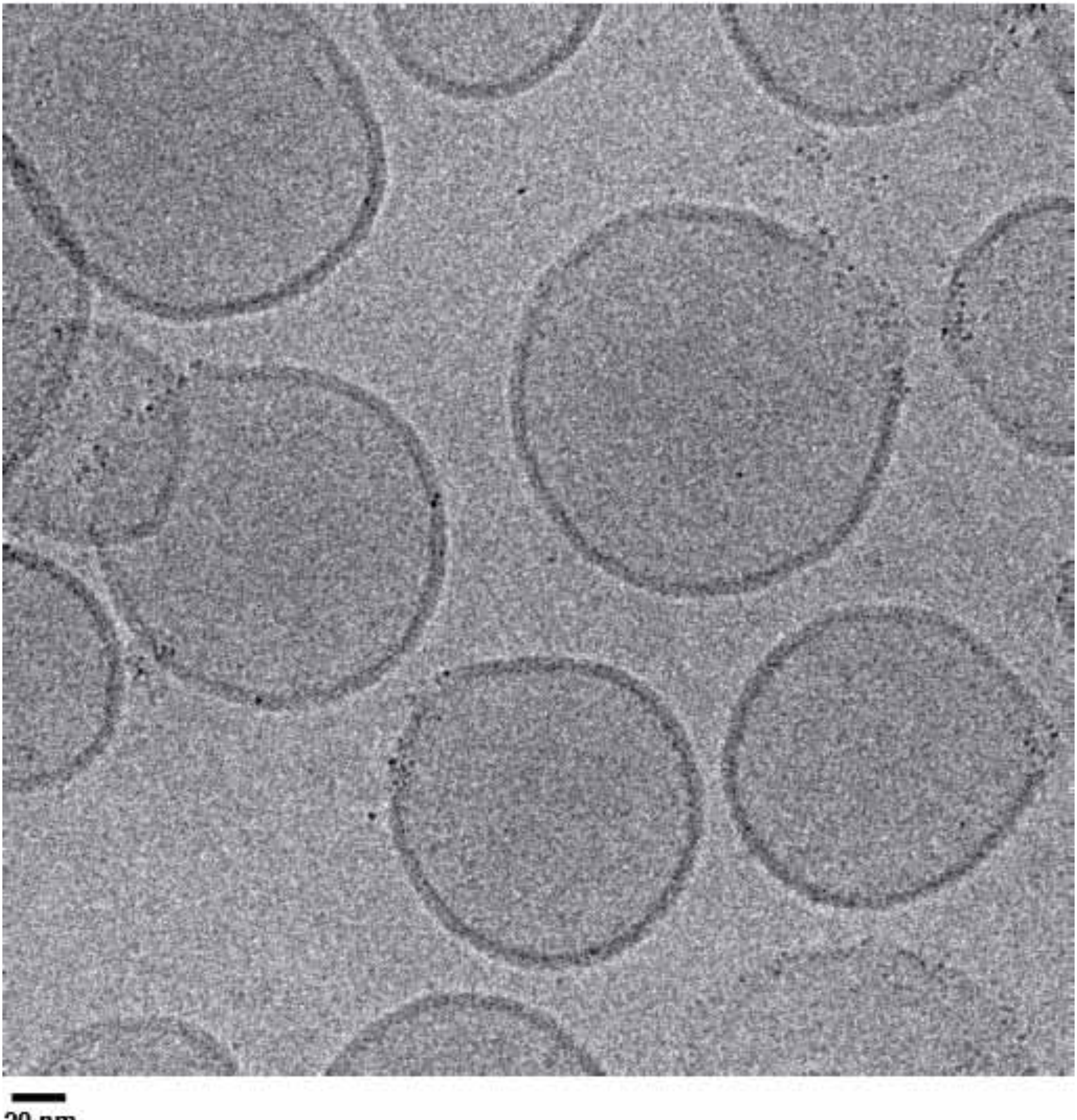

Supplemental Figure 5

a

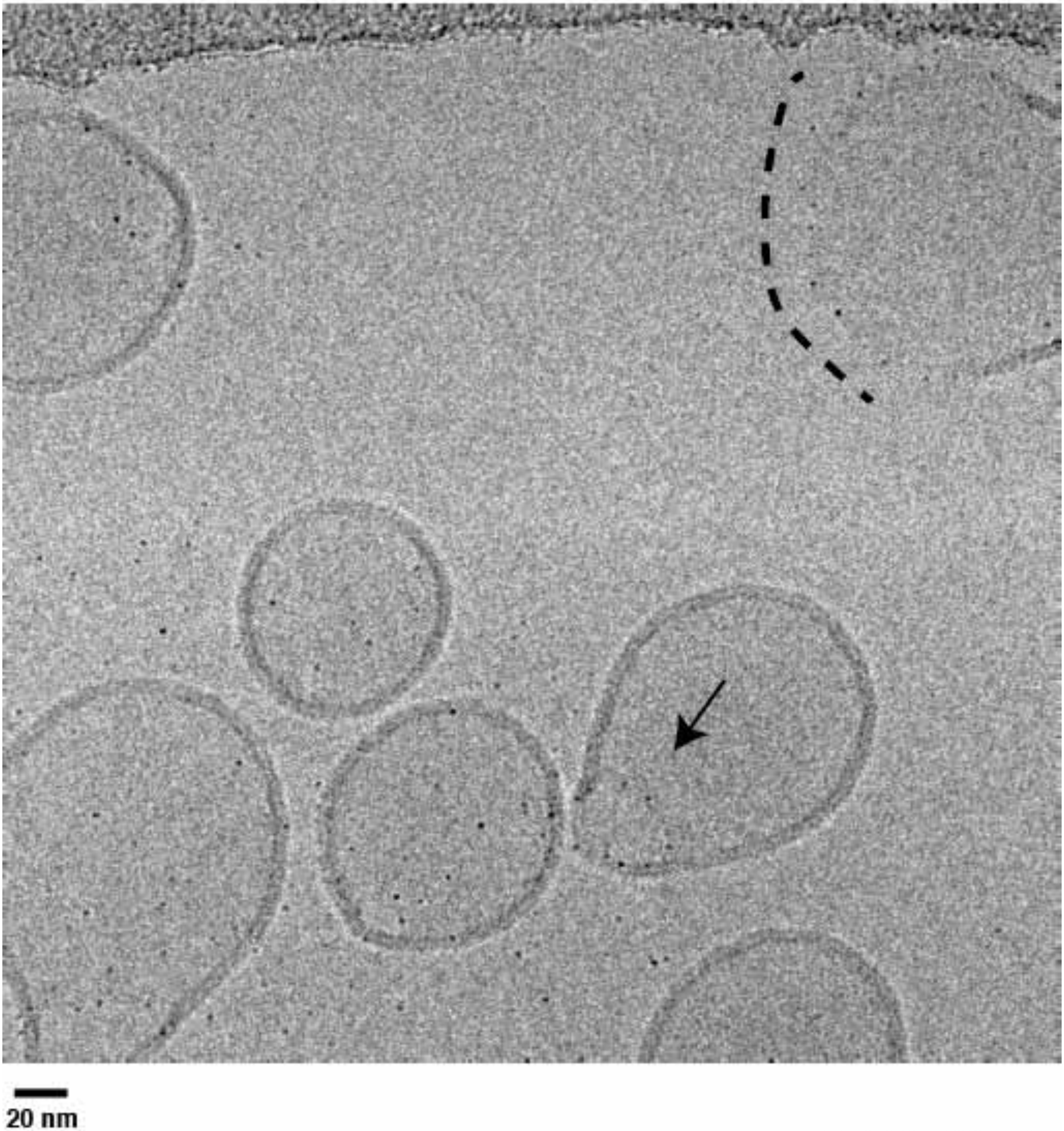

Supplemental Figure 6

b

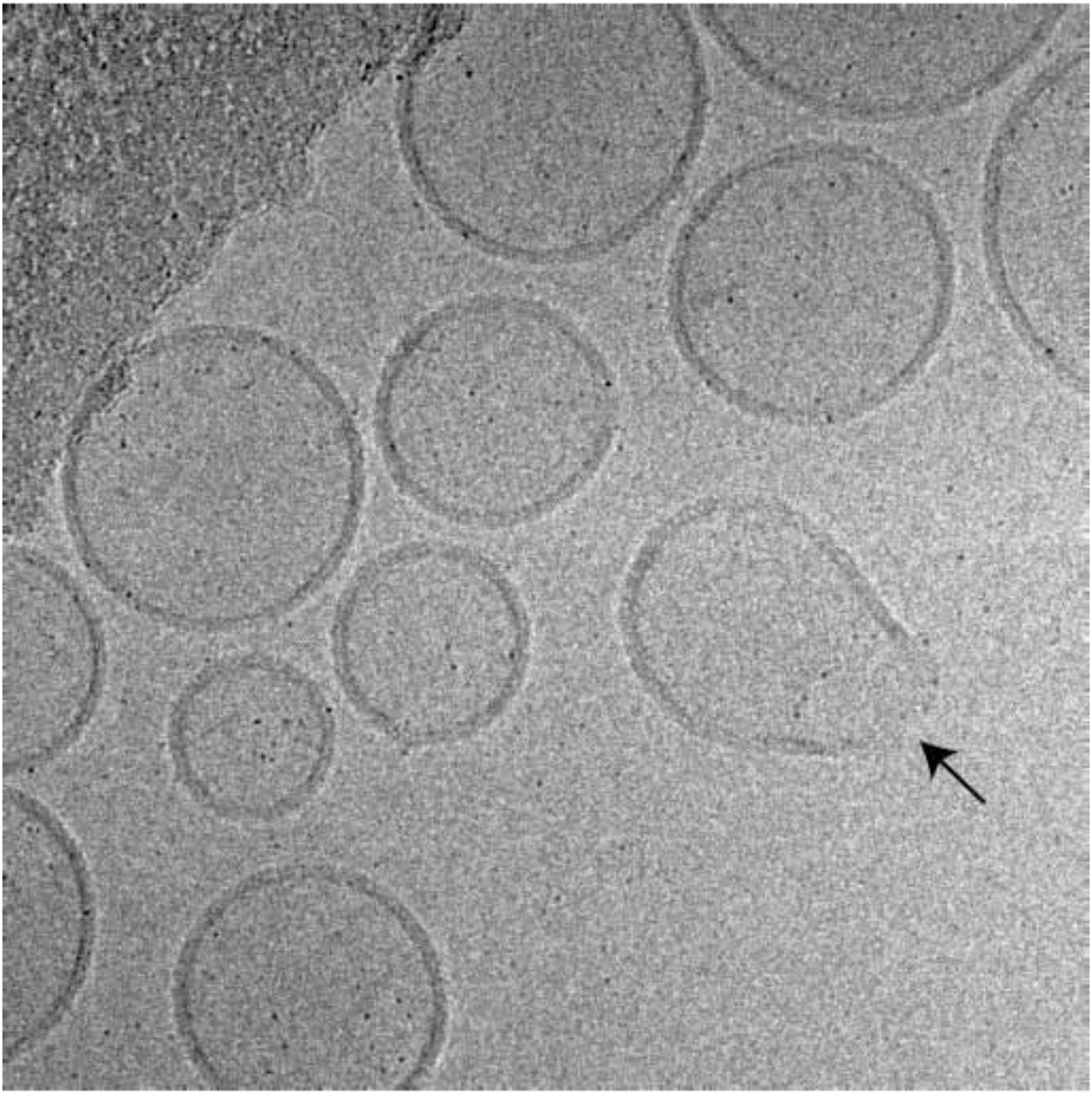

20 nm

Supplemental Figure 6

c

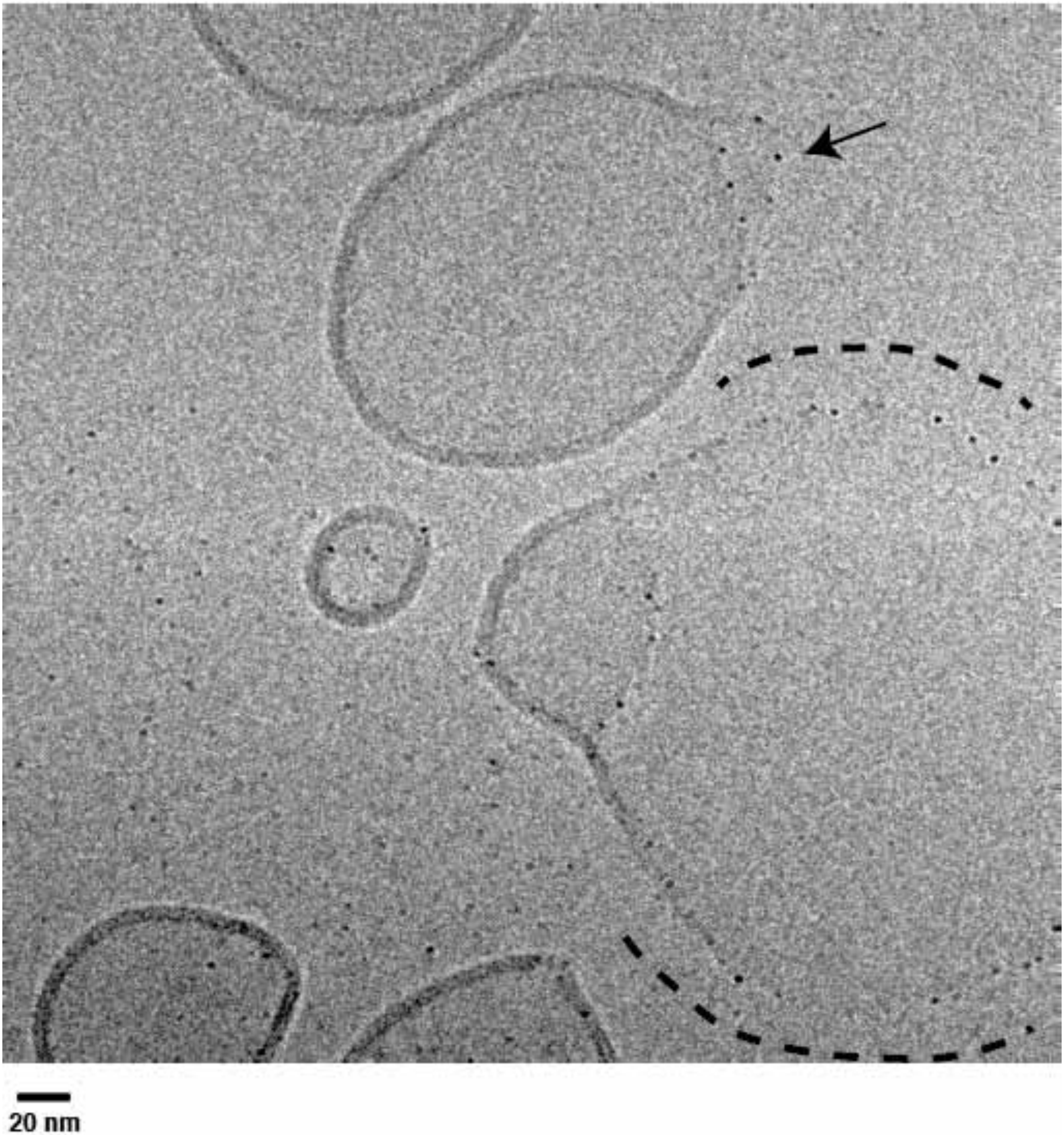

Supplemental Figure 6

Supplemental Figure 1. All Bax mutants show physiological activity

WT-Bax, gBax and His-Bax were activated by cBid (45nM) and their activations were inhibited by Bcl-xL. The kinetic dextran release assay was performed as described [16].

Supplemental Figure 2. gBax stays monomeric after concentration and frozen storage

After preparation of gBax, it was concentrated and stored frozen (fraction #6). It was thawed and fractionated again on Sephadex 75. The fractions were loaded on SDS-PAGE with non-reducing conditions and nanogold was stained with silver enhancing. The gBax appears to have peaked at between fraction #5 and #6, compared to #6 of the first fractionation. Since the gel bed was disturbed between these two runs and the standard molecular weight markers exhibit the same slight peak shift, we conclude that the peak position of gBax in the second fractionation stayed the same.

Supplemental Figure 3a-c. Three entire images taken from gBax control

OG-LUVs were permeaqbilized with WT-Bax (3.4 uM) and cBid (3.1 uM) in the presence of inactivated nanogold at 37°C for 1.5h. Large dark materials denoted in a) and seen in b) and c) also (asteriscs) are ice crystal contamination. Vesicles were well permeabilized and some of them lost their integrity, spreading like sheets. Note the edges of the pores or the fragmented sheets are rarely

decorated with nanogold particles. Note the scale bar; the nominal magnification at the scope was lower (29,000x) in this set.

Supplemental Figure 4a-c. Three entire images taken from gHis-Bax control
OG-LUVs were treated with WT-Bax (5.7 uM) and cBid (3.1 uM) at 37°C for 2 h. 1uL of Ni^{++}-nanogold was added the last 30 min as in the assay. Many edges of the pores are visible and gold particles are scattered, but association with the pore edges is not distinct.

Supplemental Figure 5a-c. Three entire images taken from gBax sample
The images show specific localizations of gold particles in one to a few regions of a vesicle. Often times, the region that gold particles cluster exhibit membrane curvature changes, possibly due to the presence of the pore. The pore edges are visible in some vesicles (arrows). There is a recurring feature of clustering of Bax (stars) on the focused regions of the membrane. We do not know what they are at the moment.

Supplemental Figure 6a-c. Three entire images taken from gHis-Bax sample
Obvious pores (also seen in Figure 2a) are indicated by arrows. In addition, some of the edges of the liposome membrane are decorated by the gold particles (dashed lines). These disintegrated or fragmented vesicles are likely a result of excessive permeabilizing effect by Bax.
